# Vascular density of optic nerve head in diabetic retinopathy using optical coherence tomography angiography

**DOI:** 10.1186/s40942-020-00269-2

**Published:** 2020-12-02

**Authors:** Fariba Ghassemi, Sahar Berijani, Ramak Roohipoor, Masoumeh Mohebbi, Ameneh Babeli, Alireza Gholizadeh, Siamak Sabour

**Affiliations:** 1 Eye research center, Farabi Eye Hospital, Tehran University of Medical Sciences, Qazvin Square, Tehran, 1336616351 Iran; 2 Retina & Vitreous Service, Farabi Eye Hospital, Tehran University of Medical Sciences, Tehran, Iran; 3 Cornea Service, Farabi Eye Hospital, Tehran University of Medical Sciences, Tehran, Iran; 4 Department of Clinical Epidemiology, School of Health and Safety, Safety Promotion and Injury Prevention Research Centre, Tehran, Iran; 5 Department of Clinical Epidemiology, Shahid Beheshti University of Medical Sciences, Tehran, Iran

**Keywords:** Capillary plexus, Choriocapillaris, Diabetes, Diabetic retinopathy, Optical coherence tomography angiography, Papillary, Peripapillary, Radial peripapillary capillaries, Vascular density

## Abstract

**Purpose:**

To measure optic nerve head (ONH) blood perfusion using optical coherence tomography angiography (OCTA) at various stages of diabetic retinopathy (DR).

**Methods:**

One hundred seventy six eyes of 94 patients included in this retrospective single-centre cross-sectional study. The subjects were studied in normal, no diabetic retinopathy (NDR), non-proliferative diabetic retinopathy (NPDR) and proliferative retinopathy (PDR) groups. The eyes were subjected to AngioDisc ONH imaging using OCTA for papillary (Disc) and peripapillary (RPC) vascular density (VD) evaluation.

**Results:**

The mean age of the participants was 56.08 ± 8.87 years and 34 (36.2 percent) were male. With increased DR severity, a statistically significant decrease in peripapillary VD was found. The study showed that only VD of the whole RPC (W-RPC) could be a valid biomarker in the staging assessment. VD of RPC, in all subsections, was considerably different from normal cases in the PDR group. Visual acuity was correlated with whole image ONH VD. The duration of DM, FBS, hyperlipidemia and DME had no effect on the ONH perfusion.

**Conclusions:**

The study showed that only the W-RPC VD could be a reasonable marker in the staging assessment. VDs assessed by OCTA can be useful for assessing and tracking early ONH changes in DR patients.

## Synopsis

At the radial peripapillary capillaries, optical coherence tomography angiography based vascular density is significantly correlated with the severity of diabetic retinopathy and decreases with its exacerbation.

## Introduction

Diabetic retinopathy (DR) can be a debilitating complication of diabetes mellitus (DM) and is a worldwide leading cause of blindness [1]. Diabetic macular edema (DME) and various forms of optic neuropathy (ONP) are other ocular complications of diabetic patients [2]. Optic neuropathies that can occur in 38.4% of DR patients include optic disc neovascularization (NVD), diabetic papillopathy (DP), non-arteritic anterior ischemic optic neuropathy (AION), and optic atrophy (OA), and some undefined nonspecific diabetic ONP [3]. Although there have been many significant advances in the understanding of the disease over the past few decades, the primary pathogenesis of DR has yet to be identified. Retina perfusion deficiency may result in DR and ONP in the course of DM. It has been shown that the duration of the DM was associated with the ONP [3]. DR is characterized by endothelial injury, a leaky blood–retinal barrier, vascular occlusion, and ischemia leading to neovascularization and DR progression [4].

An optic nerve head (ONH) is supplied by the posterior ciliary artery and central retinal artery. The radial peripapillary capillaries (RPC) as the main capillary bed are the most common capillaries located in the inner part of the nerve fiber layer around the ONH. Jia et al. have found that the relatively dense RPC spreads as far as 5.5 mm from the disc center [5].

Optical Coherence Tomography Angiography (OCTA) is a new non-invasive quantitative technique used to examine retinal and peripapillary microvasculatures [6]. The development of OCTA has enabled visualization and quantification of the RPC and papillary vessels in various retinal vascular diseases such as DR [6–10].

Few previous studies reported some microvascular changes in ONH even in subclinical phase of the disease [8–11]. It is shown that the density of the RPC and the thickness of the peripapillary retinal nerve fiber layer (PP-RNFL) were significantly reduced in subclinical DR relative to control subjects [8–11]. Little is known about the VD of papillary (disc) and PP area in advanced stages of DM. Qualitative and quantitative studies are recommended for better analysis of the pathophysiology of the disease over the course of the disease. In the absence of a database of the characteristics of VD of ONH in DM, we have been advised to do OCTA of ONH for finding a potentially useful biomarker for subclinical and clinical patients with DR. A better understanding of the neurovascular pathogenesis of DR may provide new and more effective preventive strategies.

## Methods

### Subjects

This study is a retrospective, single-centre, and cross-sectional study performed in the retina service of Farabi Eye Hospital, Tehran, Iran, between Jan 2016 and May 2020. The institutional committee of Tehran University of Medical Sciences approved this study (IR.TUMS.VCR.REC.1396.4669). The research was performed in accordance with the tenets of the Declaration of Helsinki. Informed consent was obtained from all the participants. Seventy-four patients with DM and 20 healthy controls were recruited for the study. The endocrinologist determined the diagnosis of DM, according to the diagnostic guidelines of the American Diabetes Association, and they were all under treatment [12]. Classification and diagnosis of DR were established according to the Early Treatment Diabetic Retinopathy Study (ETDRS) criteria [13].

All consecutive naive patients with a history of more than 10 years of diabetes were included and control patients were chosen from healthy volunteers. The best-corrected visual acuity (BCVA) of 20/20 for normal cases and refractive error between − 3 and + 1 D spherical equivalent in all groups were needed. Exclusion criteria included patients with other ocular diseases affecting the neural and vascular structures of the eye (glaucoma, uveitis, optic neuropathy, age-related macular degeneration, central serous chorioretinopathy or pachychoroid history, retinal artery/vein occlusion, refractive error > 3 diopters), the history of ocular surgery, ocular trauma, amblyopia, intraocular pressure (IOP) > 21 mmHg, any cardiovascular disease or systemic corticosteroid use and any media opacity precluding high quality OCTA imaging (scan quality > 6). Both eyes were used in the case of having good image quality [14–17]. The measurement maps of vascular density are based on pixels, so we need to have a high image quality so that the OCTA software does not interpret the “lack of pixels” as “low vascular density,“ when we know that high signal strength is essential for capillary identification.

Three readers (SB, AB and FG) from Image Reading Center reviewed and graded all OCT images. If the initial pictures of a patient were of low quality, we repeated the scans until it was possible to acquire a picture of at least reasonable quality. Complete ocular examination was performed, including slit lamp anterior and posterior segment examination, best-corrected visual acuity (BCVA), and intraocular pressure (IOP) measurement.

All data were obtained in the morning at the same hospital and by the same equipment and personnel. All patients underwent blood pressure measurement. Demographic characteristics and relevant laboratory tests such as fasting blood sugar (FBS) and total serum cholesterol level were documented. Hypertension under control was defined as systolic blood pressure below or equal to 140 and diastolic blood pressure below or equal to 85 mmHg in the clinic and in the sitting position. Hemoglobin A1C levels were not checked for all the cases.

The patients with diabetes were divided into three groups depending on the degree of DR [13] as no diabetic retinopathy (NDR), non-proliferative DR (NPDR), and proliferative DR (PDR).

### Image acquisition

**Fig. 1:**
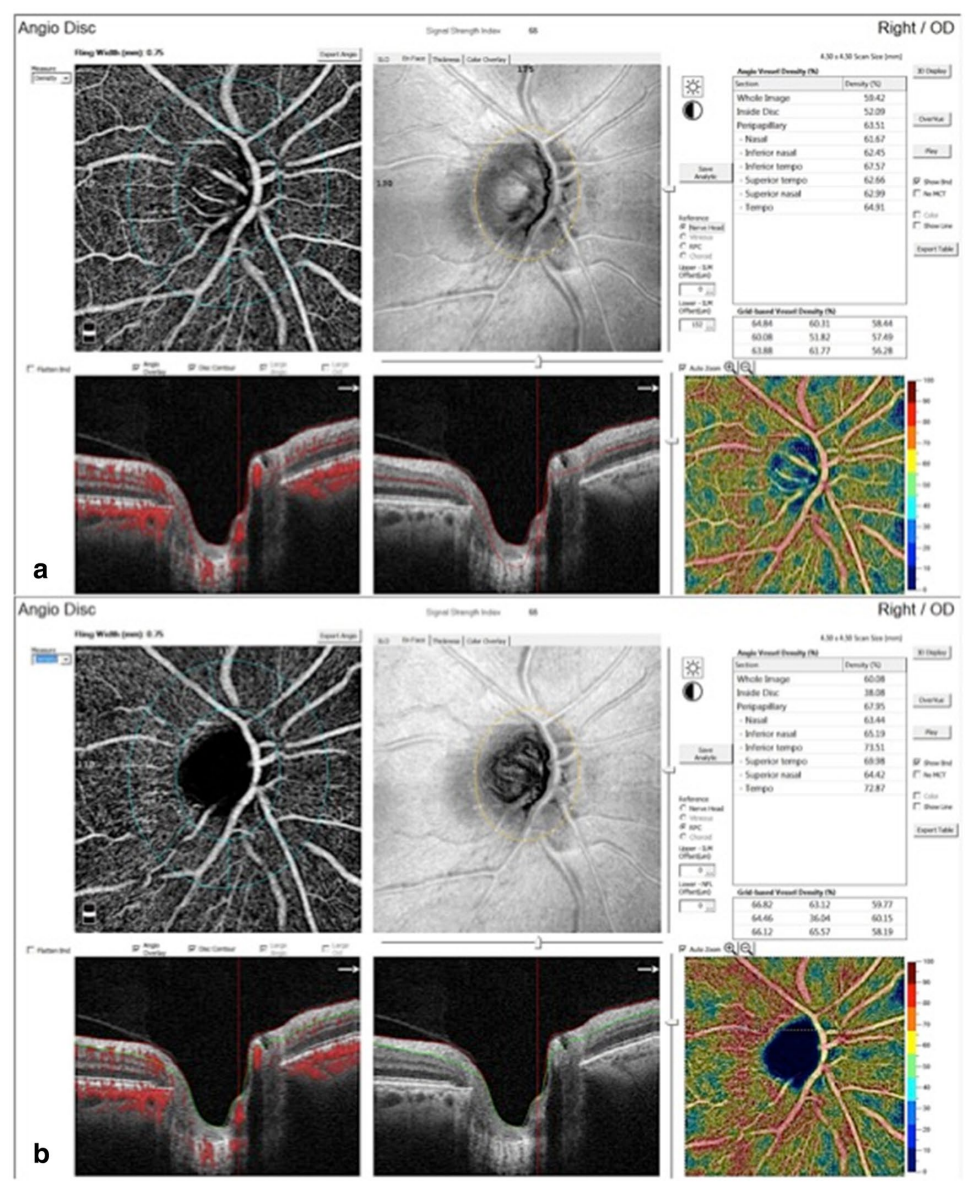
Optic nerve head optical coherence tomography angiography images (Angio Disc view) of diabetic patients at different severity scores and normal eyes. **a** At the level of disc. **b** At the level of radial peripapillary capillaries (RPC)

AngioDisc image was performed for all eyes using OCTA (RTVue XR Avanti; Optovue, USA-version: 2016.1.0.23-beta). Two volumetric raster scans, including 1 horizontal priority (x-fast) and 1 vertical priority (y-fast), were performed consecutively, within 15 seconds. Every collection of scans consisted of four default 4.5 × 4.5 mm^2^ optic disc images obtained from 32,000 OCTA axial line scans based on the optic nerve head. The split-spectrum amplitude decorrelation angiography (SSADA) algorithm analyzed the volumetric scans, and most motion artifacts were eliminated by 3D orthogonal registration and the combining of 2 scans [6]. Imaging was performed after 3 to 5 minutes of rest, with a mydriatic drop (Fig. 1) [18].

The VD of ONH was calculated using a cross-sectional–registered automated algorithm, defined as the regional percentage of blood vessels in the disc and PP area (Fig. 1). Two concentric circles with 1.5 and 3.4 mm diameter were centered on the disc area. The measurement of VD of RPC was made from the internal limiting membrane as a plane of reference to 152 µm to the interface of the inner nuclear layer and outer plexiform layer as the outer boundary, using the co-registered OCT B-scans in each eye [19]. The operator manually fine-tuned the plane to maximize the visualization of the RPC. The OCTA scan was automatically subdivided into sectors as whole image ONH (WI-ONH), whole RPC (W-RPC), whole disc (W-disc) and nasal (N), temporal (T), superonasal (SN), superotemporal (ST), inferonasal (IN), and inferotemporal (IT) subsegments on both disc and RPC areas (Fig. 1a). Change in macular thickness was also measured.

We studied the papillary flow area (mm^2^), representing the area occupied by the vessels, detected from all depths of the disc, from the top of the ONH (inner surface) to the lamina cribrosa. We automatically calculated the peripapillary VD in RPC using software that included both the RPCs and the large retinal vessels around the disc. This was thus a combination of disc and retinal circulation and not a mere calculation of a single vascular surface.

### Statistical analysis

All quantitative variables were reported as mean with standard deviation after confirming normality of distribution with the Kolmogorov–Smirnov test. Non-normal distributed parameters are reported by median with the range. All statistical analyses were performed using statistical software (SPSS software Version 21; SPSS, Inc., Chicago, IL, USA). Kruskal–Wallis test and one-way analysis of variance (ANOVA) were performed for nonparametric and parametric comparison. Mann Whitney U test and post-hoc analysis (Tuckey test) were used to pairwise comparison of the groups. Univariate and multivariate regression analysis were performed to evaluate the effect of VD predictors. To reduce collinearity, only variables with a variance inflation factor of less than 4 were included in the final model. P values less than 0.05 were considered statistically significant.

## Results

One hundred seventy six eyes of 94 patients (mean 56.08 ± 8.87 years) were studied (Table 1). Of these 34 (36.2%) were male. For PDR and NPDR patients BCVA was substantially lower compared to NDR and normal subjects. Mean FBS was 209.85 ± 85.6 mg/dl in the diabetic patients. Mean diabetes mellitus (DM) duration in diabetic patients was 12.7 ± 6.3 years. The normal control group comprised 37 eyes, and the diabetic group consisted of 139 eyes. Based on the DR severity score, the diabetic group had 33 (24.1%) eyes with NDR, 43 (31.4%) eyes with mild to moderate NPDR, 21 (15.3%) eyes with severe NPDR, 27 (19.7%) with early PDR and 13 (9.5%) eyes with high-risk characteristic PDR. Diabetic macular edema (DME) was present in 34 (58.4%) of NPDR and 18 (39.1%) of PDR patients. Table 1 presents the baseline characteristics of the participants.

**Table 1:** Optic nerve head and peripapillary superficial vascular density and flow of eyes at various diabetic stages compared to normal eyes (N = 176)

Groups	NL (37 eyes) (M ± SD)	NDR (35 eyes) (M ± SD)	NPDR (58 eyes) (M ± SD)	PDR (465 eyes) (M ± SD)	p-value
Age (years)	53.0 ± 0.9	59.0 ± 1.1	56.0 ± 1.1	57.1 ± 1.1	0.074
(%) OD	19 (51.4)	17 (48.6)	37 (53.4)	23 (50)	0.971
Sex-male (%)	23 (62.2)	11 (31.4)	21 (36.2)	21 (45.7)	*0.034*
BCVA (decimal)	0.98 ± 0.00	0.91 ± 0.01	0.62 ± 0.03	0.64 ± 0.04	*< 0.001*
LOG MAR	0.02 ± 0.00	0.09 ± 0.01	0.42 ± 0.13	0.52 ± 0.06	*0.001*
The last FBS (mg/dl)	All < 100	163.8 ± 6.5	221.2 ± 9.8	231.52 ± 12.0	*< 0.001*
Duration of diabetes mellitus (years)	–	10 ± 1.1	11.8 ± 0.7	15.5 ± 0.9	*< 0.001*
Hypertension number (%)	2 (5.4%)	15 (46.9%)	26 (46.4%)	14 (48.4%)	*< 0.001*
Hyperlipidemia number (%)	0 (05%)	12 (37.5%)	27 (48.2%)	17 (58.6%)	*< 0.001*

*P* values less than 0.05 were considered statistically significant

BCVA: Best corrected visual acuity; DME: Diabetic macular edema; FBS: Fasting blood sugar; NDR: No diabetic retinopathy; NL: Normal; NPDR: Nonproliferative diabetic retinopathy; PDR: Proliferative diabetic retinopathy

The difference in BCVA and sex were statistically significant. Spherical equivalent, axial length or IOP did not vary significantly in the groups (p > 0.05). Hyperlipidemia and hypertension were more prevalent in the past medical history of diabetic groups than the control group (p < 0.001). The hypertension was under control in all patients.

### Vascular densities

The VD of WI-ONH, the W-disc VD and the W-RPC were evaluated between two normal and diabetics groups. The WI-ONH VD was 55.64 ± 3.87 percent for the diabetes group and 57.80 ± 2.68 percent for the control group (p = 0.002). W-disc VD was 51.41 ± 5.98 percent in the diabetes group and 53.04 ± 4.15 percent in the control group (p = 0.122). The VD of W-RPC was 59.26 ± 4.42 percent in diabetic groups and 62.26 ± 2.78 percent in the control group (p < 0.001) (Table 2).

**Table 2:** Vascular density in optic nerve head (ONH) of normal subjects and the diabetic patients (N = 176)

Groups VD/ Reference group	NL (37 eyes) Mean (%)	DR (138 eyes) Mean (%)	p value
Whole image	57.80 ± 2.68	55.64 ± 3.87	*0.002*
Whole disc	53.04 ± 4.15	51.41 ± 5.98	0.122
N-disc	51.78 ± 6.66	52.4 ± 7.9	0.657
IN-disc	56.82 ± 10.85	56.75 ± 2.68	0.972
IT-disc	53.16 ± 6.56	48.99 ± 9.97	*0.017*
ST-disc	52.11 ± 8.37	49.99 ± 9.46	0.218
SN-disc	57.12 ± 7.74	57.83 ± 8.97	0.661
T-disc	50.55 ± 6.80	45.71 ± 8.05	*0.001*
Whole RPC	62.26 ± 2.78	59.26 ± 4.42	*< 0.001*
N-RPC	60.53 ± 3.90	57.43 ± 6.05	*0.004*
IN-RPC	63.86 ± 3.37	60.14 ± 6.44	*0.001*
IT-RPC	65.62 ± 3.53	61.64 ± 5.89	*< 0.001*
ST-RPC	63.03 ± 4.44	60.69 ± 5.31	*0.015*
SN-RPC	61.81 ± 4.32	58.89 ± 7.37	*0.023*
T-RPC	61.95 ± 3.76	59.48 ± 4.82	*0.004*
PP blood CC flow-2 mm radius	7.56 ± 0.20	7.32 ± 0.81	0.089
PP blood CC flow- /area^2^ mm radius	0.60 ± 0.01	0.59 ± 0.01	*0.009*

*P* values less than 0.05 were considered statistically significant

Mann–Whitney test

In the post-HOC analysis the reference groups were stated in the rows

CC: Choriocapillary; IT: Inferonasal; IT: Inferotemporal; NDR: No diabetic retinopathy; N: Nasal; NL: Normal; NPDR: Nonproliferative diabetic retinopathy; PDR: Proliferative diabetic retinopathy; PP: Peripapillary; RPC: Radial peripapillary capillary; SN: Supreronal; ST: Superotemporal; T: Temporal

**Fig. 2:**
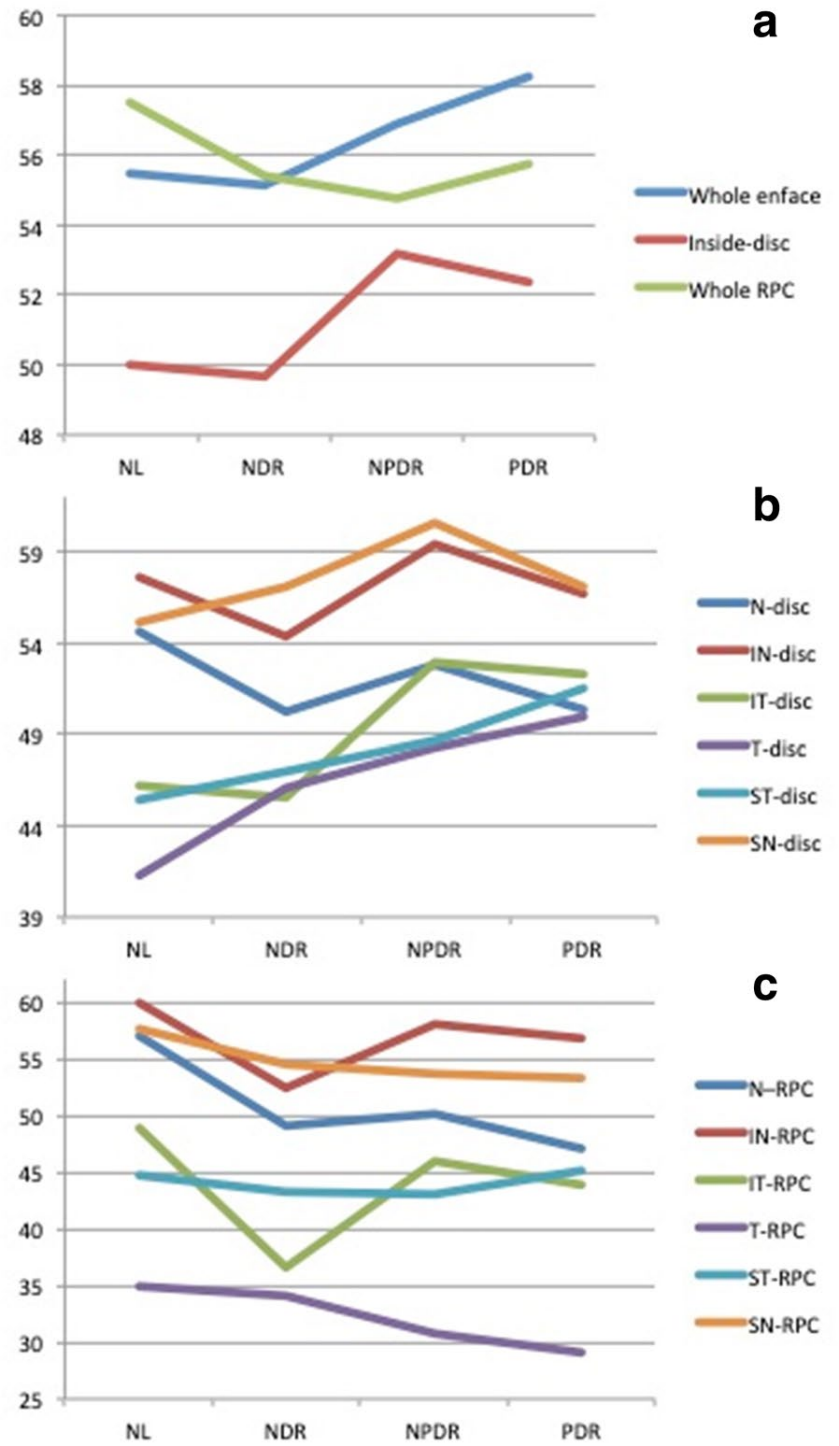
Vascular density of optic nerve head using on OCTA images using Angio Disc view of diabetic patients at different severity scores and normal eyes. **a** Whole image, disc and peripapillary vascular density. **b** The segmental vascular density on the disc. **c** The segmental vascular density on the radial peripapillary capillary level

A statistically significant decreasing order in VD was found in the WI-ONH, W-disc and W-RPC among the four groups (p < 0.05) (Table 3; Fig. 2). Of various subsegments of the disc itself, only the VD of IT and T tended to decrease, mostly during the PDR stage. In post-hoc analysis, the VD decrease of these two locations compared to normal cases was statistically significant (Table 3). In the RPC, in all but the N and IN subsegments, the VD has decreasing order while increasing the disease severity. Post-hoc analysis showed that VD of RPC at all subsegments was significantly different from normal cases in PDR group. It seems that in the RPC region, only W-RPC VD could be a reliable biomarker in the assessment of staging. Only in the W-RPC and N-RPC, the decrease in VD was statistically significant in the NDR group relative to the normal group.

**Table 3:** Vascular density in optic nerve head (ONH) of normal subjects and subtypes of diabetic retinopathy (N = 176)

Groups VD/ Reference group	NL (37 eyes) Mean ± SD (%)	NDR (35 eyes) Mean ± SD (%)	NPDR (58 eyes) Mean ± SD (%)	PDR (46 eyes) Mean ± SD (%)	p-value Kruskal–Wallis test
Whole image (median) NL NDR NPDR	58.26 (53.54–61.60)	56.90 (49.73–61.62) 0.145*	55.11 (46.48–59.59) 0.014* 0.398*	55.48 (49.94–55.48) < 0.001* 0.018* 0109*	*0.002*
Whole disc NL NDR NPDR	52.39 ± 0.96	53.15 ± 0.96 1.000	49.66 ± 1.90 0.746 0.767	50.03 ± 1.62 *0.033* *0.039* 0.191	*0.018*
N-disc NL NDR NPDR	50.35 ± 1.87	52.76 ± 1.37 0.958	50.23 ± 2.50 0.862 0.996	54.66 ± 2.30 0.995 0.869 0.683	0.684
IN-disc NL NDR NPDR	56.74 ± 3.14	59.35 ± 2.10 0.582	54.33 ± 3.81 0.993 0.345	57.66 ± 2.24 0.833 0.136 0.913	0.182
IT-disc NL NDR NPDR	52.35 ± 1.44	52.91 ± 1.94 0.869	45.51 ± 3.04 0.130 0.560	46.15 ± 2.75 *0.024* 0.823 0.823	*0.022*
T-disc NL NDR NPDR	49.91 ± 1.80	48.34 ± 1.36 0.498	46.05 ± 2.05 0.145 0.945	41.25 ± 1.78 *< 0.001* *0.004* *0.006*	*< 0.001*
ST-disc NL NDR NPDR	51.47 ± 2.18	48.66 ± 2.11 0.648	46.92 ± 2.04 0.962 0.850	45.42 ± 2.70 0.463 0.997 0.679	0.426
SN-disc NL NDR NPDR	57.13 ± 2.20	60.59 ± 2.25 0.419	57.10 ± 2.16 0.999 0.398	55.13 ± 2.45 0.996 0.260 0.981	0.272
Whole RPC NL NDR NPDR	62.24 (56.78–67.02)	59.68 (55.13–65.35) *0.006*	60.07 (50.83–64.37) 0.009* 0.991*	59.35 (51.42–65.48) < 0.001* 0.036* 0.041*	< 0.001*
N-RPC NL NDR NPDR	60.07 (53.26–67.12)	57.33 (50.30–64.84) < 0.001*	59.41 (36.69–53.39) 0.087* 0.631*	58.66 (44.94–66.08) 0.001* 0.112* 0.073*	0.011*
IN**-**RPC NL NDR NPDR	53.75 ± 1.00	60.22 ± 1.26 < 0.104	60.31 ± 2.01 *0.031* 0.997	60.81 ± 1.28 *0.006* 0.836 0.884	*0.0001*
IT-RPC NL NDR NPDR	64.48 ± 0.92	63.64 ± 0.75 0.331	60.94 ± 1.21 *0.006* 0.486	61.11 ± 1.04 *< 0.001* *0.037* 0.427	*< 0.001*
T-RPC NL NDR NPDR	62.45 ± 0.78	60.84 ± 0.73 0.648	58.95 ± 0.70 0.911 0.911	58.00 ± 1.86 *< 0.001* *0.030* 0.072	*0.001*
ST-RPC NL NDR NPDR	62.63 ± 1.07	61.45 ± 1.11 0.622	59.66 ± 2.16 0.993 0.993	55.13 ± 2.45 *0.006* 0.196 0.206	*0.012*
SN-RPC NL NDR NPDR	62.34 (49.65–47.35)	59.06 (50.16–59.06) 0.099	60.38 (55.23–68.06) 0.087 0.686*	58.46 (35.64–68.39) *0.016* 0.336* 0.517*	*0.047*
Peripapillary CC flow (median) NL NDR NPDR	7.57 (7.19–7.89)	7.63 (6.81–7.84) 0.890*	7.50 (1.74–8.80) 0.023* 0.024*	7.43 (1.5–7.79) 0.003* 0.004* 0.283*	*0.003*
Peripapillary CC flow- /area^2^ mm radius NL NDR NPDR	0.61 (0.58–0.63)	0.60 (0.56–0.62) 0.713*	0.60 (0.52–0.62) 0.009* 0.016*	0.59 (0.50–0.63) 0.001* 0.003* 0.482*	*0.001*

*P* values less than 0.05 were considered statistically significant

In the post-HOC analysis the reference groups were stated in the rows

NDR: No diabetic retinopathy; NL: Normal; NPDR: Nonproliferative diabetic retinopathy; PDR: Proliferative diabetic retinopathy

*Mann–Whitney test

When the area of choroidal vasculature flow was assessed, choroidal vasculature decreased by increasing of DM severity (Table 2). The VD of ONH area were not correlated with FBS and duration of DM in diabetic patients.

### BCVA

In the ONH area, BCVA was correlated with VD of WI-ONH (r = 0.307, p = 0.045), W-disc (r = 0.307, p = 0.045), IT-RPC (r = 0.37, p = 0.014), and IT-disc VD (r = 0.37, p = 0.014) in univariate analysis of diabetic group. A model of multivariate lineal regression showed that only WI-ONH VD was directly correlated with BCVA (B = 0.266, p = 0.03). This means that BCVA will decrease by 2.6 lines (decimal) for every 10% decrease in WI-ONH density.

### FBS

FBS was correlated with VD of IT-RPC (r = 0.434, p = 0.003) and ST-disc (r = − 0.328, p = 0.03) in univariate analysis. In a multivariate linear regression analysis none of them was important after covariate adjustment.

### Duration

The duration of diabetic retinopathy was not correlated with any of the evaluated parameters at ONH in univariate and multivariate analysis.

### DME

The presence of DME was associated with less amount of VD in diabetic patients compared with those without DME as WI-ONH VD (56.4% vs. 54.6%, p = 0.011), W-disc (52.8% vs. 49.9%, p = 0.005), SN-disc (59.7% vs. 55.3%, p = 0.007), T-disc (47.6% vs. 43.5%, p = 0.006), IT-disc (50.7% vs. 47.1%, p = 0.05), W-RPC (60.1% vs. 57.1%, p = 0.015), N-RPC (69% vs. 52%, p = 0.021), and T-RPC (65.5% vs. 58%, p = 0.006). In multivariate regression analysis none of these segments VD were correlated with DME. This may mean that the occurrence of DME was independent of the ONH perfusion.

## Discussion

The following results have been documented in this study.

1. In the W-RPC and all sub-segments except the N-RPC and IN-RPC, the VD has a decreasing order while increasing the severity of the disease. The VD of the W-RPC could be a good biomarker in the assessment of staging of DR.
2. The perfusion of disc was not affected by the severity of the disease except at IT-disc and T-disc segments, which decreased mainly during the PDR stage.
3. The frequency of DME was independent of the ONH perfusion.
4. The FBS and DM duration do not directly affect ONH perfusion.

To date, only a few studies have used OCTA to determine changes in ONH microcirculation in DR [8, 20]. In our research, PP perfusion was examined segmentally in patients with different DR stages with OCTA.

In agreement with other studies, our study showed that ONH capillary density was significantly reduced in the DM group compared to the control group [8]. Unlike other reports, we have shown that inside the disc, changes are only in temporal and inferotemporal areas, but in the PP region, all segments have less measure in diabetics. The amounts observed for the disc and RPC VD in this study are higher than other researches that may be attributed to ethnic variation or imaging technique [8].

The results are in accordance with other studies, showing that the RPC is less in NDR compared with the control group, [10, 21] but the amounts are only statistically significant at W-RPC and N-RPC.

Unlike the analyzing method of the RPC used by Rodrigues [9], Vujosevic [10], and Shin,[21] our study analyzed the ONH area by segments. The number recorded by Shin et al. is much less than ours, which is possibly due to using the 6 × 6 mm scale and the OMAG algorithm analyzes by Zeiss Cirrus HD-OCT 5000 instrument in their study [21].

Leukostasis and slow circulation in the diabetic retina may cause hypoxia at some point [22–24] and the excitotoxins damage to glial cells [25] leads to impaired neurovascular coupling and hypoperfusion [26], and the loss of endothelial cells and pericytes could destroy capillaries [27]. Such perfusion deficit mechanisms may be the source of the observed findings.

In agreement with our previous report [6] in the present study, we found that the IT-RPC sector had the highest VD and T-disc the lowest VD in normal subject. This finding has also been identified more or less in all three diabetic groups. Hafner et al. reported a pattern of higher temporal than nasal flow of the ONH, statistically significant differences between the four quadrants could not be observed [28]. They disclosed that a mal-distribution of oxygen occurs even in patients with NDR [28].

In some OCTA reports, the VD of ONH and PP retina were significantly reduced in glaucoma patients, which is associated with RNFL thickness and loss of visual field in glaucoma patients [29–31]. DM is a well-known risk factor for primary open-angle glaucoma [32, 33].

Nicola et al. reported less W-disc and PP area VD in central retinal vein occlusion (CRVO) than normal fellow eyes, which are significantly increased after treatment [34]. Another study showed that mean RNFL thickness and W-RPC VD were significantly lower in the non-arteritic anterior ischemic optic neuropathy (NAION) group compared to normal group [35]. Patients with retinitis pigmentosa showed a decreased ONH VD compared with healthy subjects [36]. In the present study in all segments VD measurements are significantly less in DM than normal control.

Theoretically, in each of these conditions, vascular dropout in ONH area could be primary or secondary to retinal ganglion cell complex (GCC) or loss of RNFL. In the presence of potential direct vascular damage, it is hypothesized that reduced retinal metabolic activities secondary to the reduction of retinal neurons (mainly those in the RNFL and GCC) could influence retinal perfusion through neurovascular metabolic feedback as the one in the peripapillary RNFL [37, 38]. In addition to the vascular deficit that occurs during DR even before clinical signs of full-blown retinopathy, some retinal vascular remodeling may occur. This change can be primary or secondary to axonal degeneration itself.

After adjusting for all possible recorded confounders in this study, it was shown that only WI-ONH VD was directly correlated with BCVA. In this analysis, a 2.6-line decrease in BCVA (decimal) for every 10% decrease in WI-ONH density was predicted. However, Shin et al. did not find a correlation between BCVA and the average peripapillary VD and perfusion density [21]. Li et al. reported that in the NPDR group, BCVA showed a significantly negative correlation with VD of W-disc [39].

In this study, FBS and duration of DM did not affect ONH perfusion in diabetic patients. An experimental study showed that after overnight fasting, 2 h after an oral glucose tolerance test-induced acute hyperglycemia narrowing of retinal veins and an increased arteriovenous diameter ratio occurred [40].

According to this report, the DME frequency was independent of the ONH perfusion. To the best of our knowledge, no prior study has addressed the possible correlation of DME with the ONH perfusion. Although a recent study showed that the RNFL thickness generally increases in diabetic patients with DME, and this increment is correlated with the degree of macular edema [41]. As a pure descriptive study, their results were reported without controlling for possible confounders.

While few studies have shown reduced choroidal thickness in diabetic eyes in either submacular or peripapillary areas [38] no peripapillary CC vascular flow analysis has been conducted. Our analysis revealed a steady decline in the choroid blood flow area due to increase in the severity of DR. These flow reduction could be resulted from a reduced choroidal VEGF secretion [41–44] and/or the chloride channels defect in RPE cells which can regulate the choroid vasculature [45, 46].

The strength of our study is to recruit the naïve diabetic patients. We are aware that this research has some limitations, which must be considered when assessing the results. Our research is only a cross-sectional study and is focused on a fairly homogeneous Iranian population, which precludes generalizability to other populations. The smoking habits/history and refractive errors could not be evaluated, since they were missing from some medical records. On the other hand, there is no standardization of the perfusion values of the optical disc, stratified by age and sex among others, despite the use of a control group for comparison, as exists, for example, in the databases for retinal nerve fiber layer measurements for glaucoma evaluation. Since the control group is small and may not be representative of a population, the lack of this standardization limits the comparison.

## Conclusions

OCTA findings from the ONH area could provide evidence that microvascular ONH changes occur in the early stages of DR prior to structural impairment. In AngioDisc imaging of OCTA, W-RPC may be a more promising tool in DR screening and staging. Interestingly, the occurrence of DME, FBS, and DM duration were not correlated to the perfusion of ONH. Interpolation of group study results to individuals could be inaccurate and personal prospective longitudinal studies are needed to study the actual changes at each stage.

## Data Availability

The data that support the findings of this study are available from the authors upon reasonable request and after permission of Farabi Eye Hospital managing group and research center.

## Abbreviations

Anti-VEGF: Anti-vascular endothelial growth factor
BCVA: Best-corrected visual acuity
CRT: Central retinal subfield thickness
DM: Diabetes mellitus
DR: Diabetic retinopathy
FBS: Fasting blood sugar
OCT: Optical coherence tomography angiography
ONH: Optic nerve head
RPC: Radial peripapillary capillaries
VA: Visual acuity
